# CARE COORDINATION INTERVENTIONS FOR DEMENTIA CAREGIVERS: AN INTEGRATIVE REVIEW

**DOI:** 10.1093/geroni/igad104.3209

**Published:** 2023-12-21

**Authors:** Diana Layne, Ayaba Logan, Kathleen Lindell

**Affiliations:** Medical University of South Carolina, Charleston, South Carolina, United States; Medical University of South Carolina, Charleston, South Carolina, United States; Medical University of South Carolina, Charleston, South Carolina, United States

## Abstract

Alzheimer’s disease is a serious illness with an extended caregiving experience however care coordination interventions often lack integration of palliative care. The aim of this integrative review is to identify existing care coordination interventions which integrate palliative care for community dwelling individuals with dementia and their caregivers. An integrative review guided by the Whittemore and Knafl framework was conducted, and the Mixed Methods Appraisal Tool was utilized to assess study quality. The systematic search was initially conducted in June 2021 and repeated in May 2023 to ensure no additional studies were available in collaboration with a medical informationist. Nine interventions across eighteen publications were identified that incorporated informal family caregivers and were aimed at improving care coordination for community dwelling PLWD and their caregivers. Only a single intervention used the term palliative care while the remaining interventions included traditional components of palliative care such as advance care planning, symptom management, and emotional support. Many existing care coordination interventions are not grounded in theory or conceptual models and have been studied in a homogenous sample which is not representative of the population. Further study of the existing interventions in a more heterogenous sample is needed. Additional research is necessary to better understand the lived experience of those persons with dementia and their caregivers to reduce burden associated with care coordination activities.

